# Gga-miR-205a Affecting Myoblast Proliferation and Differentiation by Targeting CDH11

**DOI:** 10.3389/fgene.2018.00414

**Published:** 2018-10-09

**Authors:** Zhijun Wang, Hongjia Ouyang, Xiaolan Chen, Jiao Yu, Bahareldin A. Abdalla, Biao Chen, Qinghua Nie

**Affiliations:** ^1^Department of Animal Genetics, Breeding and Reproduction, College of Animal Science, South China Agricultural University, Guangzhou, China; ^2^Guangdong Provincial Key Lab of Agro-Animal Genomics and Molecular Breeding and Key Laboratory of Chicken Genetics, Breeding and Reproduction, Ministry of Agriculture, Guangzhou, China; ^3^National-Local Joint Engineering Research Center for Livestock Breeding, Guangzhou, China; ^4^College of Animal Science and Technology, Zhongkai University of Agriculture and Engineering, Guangzhou, China

**Keywords:** miR-205a, CDH11, myoblast, myogenesis, promoter active region

## Abstract

Non-coding RNAs especially miRNAs have been found to play important roles during skeletal muscle development. Our previous RNA-Seq performed on breast muscle tissue from 7 weeks old Recessive White Rock and Xinhua Chicken and leg muscle tissue from female Xinghua Chicken at three development time points (11 embryo age, 16 embryo age, and 1 day post hatch) (accession number GSE62971 and GSE89355, respectively) showed that miR-205a and *CDH11* were differentially expressed genes. In this study, we found that overexpression of *CDH11* significantly facilitated Quail muscle clone (QM7) and chicken primary myoblast (CPM) proliferation and hampered CPM differentiation. MiR-205a can directly binding to the 3′UTR of CDH11 and the overexpression of miR-205a could inhibit both cell lines (QM7) and CPM proliferation, at the meantime promote the differentiation of myoblasts. The Dual-Luciferase Reporter Assay results and qRT-PCR results showed that myogenin (MyoG) could regulate the expression of miR-205a by binding to the active region of miR-205a. Altogether our data suggest that MyoG could stimulate miR-205a expression to suppress CDH11, which promotes myoblasts proliferation while represses the differentiation.

## Introduction

MicroRNAs (miRNAs) are small non-coding RNA of 20–25 nucleotides that widely participate in chicken embryo growth and skeletal muscle development (Darnell et al., 2006; van Rooij et al., 2008; Kang et al., 2013). In our preliminary study, we have used high-throughput RNA sequencing to study breast muscle transcriptome in Recessive White Rock (fast-growing chicken) and Xinghua chicken (slow-growing chicken). Another high-throughput RNA sequencing was performed in leg muscles of female Xinghua chickens at 11 embryo age (E11), 16 embryo age (E16) and 1 day post hatch (D1). These databases of RNA-Seq were aimed to find miRNAs involved in skeletal muscle development at embryonic stage. Both results pointed out that miR-205a and *CDH11* could be used as candidate genes associating with broiler growth (Ouyang et al., 2015; Jebessa et al., 2018).

Gga-miR-205a can be processed to its precursor miRNA with a mature sequence of 22 nucleotides. Since miR-205 is highly conserved among vertebrates (Wu et al., 2009), most of its target genes can overlap with human’s. MiR-205 is generally considered to be a tumor suppressor involved in the physiological processes of some cancer cells in human, for example, miR-205 can inhibit the proliferation of prostate cancer cells (Majid et al., 2010), renal cancer cells (Majid et al., 2011), and melanoma cells (Dar et al., 2011). However, the regulatory transcription factors and the way of regulating the body may be different due to the diversity of species. MiR-205a showed a high-level expression in endoderm and ectoderm during chick embryo development (Darnell et al., 2006), so we wonder its function and mechanism in muscle development combined with our previous RNA sequencing results.

*Cadherin-11* (*CDH11*) is a member of cadherin superfamily that encodes a type II classical cadherin and has a specific function in bone development and maintenance (Luo et al., 2005). Reports showed that CDH11 is a crucial regulator of postnatal skeletal growth, bone mass maintenance and osteoblast differentiation (Shin et al., 2000; Kawaguchi et al., 2001; Di Benedetto et al., 2010). The up-regulated of CDH11 could be acted as part of the response to injury in smooth muscle cell (SMC). Conversely, inhibition of CDH11 decreased SMC migration and proliferation and led to intimal hyperplasia (Monahan et al., 2007). CDH11 can be detected in restricted regions of the basal plate of the spinal cord during chicken embryo developmental stages, which indicates that CDH11 could play multiple and diverse roles during the development of the spinal cord and its surrounding tissues (Lin et al., 2014). To the best of our knowledge, some studies have focused on the function of *CDH11* in the bone formation (Kawaguchi et al., 2001; Lorda-Diez et al., 2014), however, little is known about the regulatory role of CDH11 in myoblasts.

In this study, we investigated the function and regulation of miR-205a in avian skeletal muscle development. We found that miR-205a is regulated by myogenin (MyoG) transcription factor, which can bind to the promoter region of the gga-miR-205a gene. The up-regulation of miR-205a can inhibit myoblast proliferation and promote myoblast differentiation by its repression on CDH11.

## Materials and Methods

### Ethics Standards

All animal experimental protocols in this study were carried out according to the rules and policies formulated by the committee and in accordance with the Animal Protection Law of the People’s Republic of China and approved by the Animal Care Committee of South China Agricultural University (Approval number: SCAU#0014).

### Animals

Three female chickens’ leg muscle tissues at each stage from E10 to E20 were obtained from the Chicken Breeding Farm of South China Agricultural University (Guangzhou, China), which were used to detect the expression of miR-205a in the process of chicken embryonic development.

### Primers and Plasmids Construction

All primers were designed using Premier Primer 5.0 software (Premier Biosoft International, Palo Alto, CA, United States), and synthesized by Sangon Biotech (Shanghai, China).

PmirGLO dual-luciferase reporters and gene overexpression vector: The 3′UTR fragment of *CDH11* (NCBI Reference Sequence: NM_001004371.1) containing the miR-205a binding sites were artificially synthesized by GeneCreate Biological Engineering (Wuhan, China) along with the mutation vector. The full *CDH11* coding sequence was also synthesized by the same company below and was cloned into the pcDNA3.1 vector. The full length of *MyoG* coding sequence was cloned into pcDNA3.1 vector through PMD^TM^-18T cloning vector (Takara, China), and the primers are listed in **Table 1**.

**Table 1:** Primers used for vector construction.

Primer name	Primer sequences (5′ to 3′)	Size (bp)
PGL3-basic-P1	F: CTAGCTAGCAACAGTATTTGCCACTCCTTG	727
	R: CCGCTCGAGATGGGAATCCTCTATGCTGAAACT	
PGL3-basic-P2	F: CTAGCTAGCGGAGTGGTTAATAGCTGGAAG	1320
	R: CCGCTCGAGATGGGAATCCTCTATGCTGAAACT	
PGL3-basic-P3	F: CTAGCTAGCCTCATTAGAAGGCAGAACAACA	1571
	R: CCGCTCGAGATGGGAATCCTCTATGCTGAAACT	
PGL3-basic-P4	F: CTAGCTAGCCTCACGCTCAGTTCCAGG	2006
	R: CCGCTCGAGATGGGAATCCTCTATGCTGAAACT	
PGL3-basic-P5	F: CTAGCTAGCGTGAGGTTACGGATGCTGA	2527
	R: CCGCTCGAGATGGGAATCCTCTATGCTGAAACT	
PGL3-basic-P6	F: CTAGCTAGCTGCTTGGTGGGAATGCTG	2899
	R: CCGCTCGAGATGGGAATCCTCTATGCTGAAACT	
PGL3-basic-P7	F: CTAGCTAGCTGCTGTTCCGCTTGCTCT	3223
	R: CCGCTCGAGATGGGAATCCTCTATGCTGAAACT	
PGL3-basic-P23-WT	F: TTAGAAGGCAGAACAACAA	237
	R: CTGCCAGTGGAGAAAGAA	
PGL3-basic-P23-MT	F: TTTTGAGTTAACGGTATTGGTTTTTTTATGTAAGA	2929
	R: AAAAACCAATACCGTTAACTCAAAATACAAGCTCT	
PGL3-basic-P45-WT	F: GGTTACGGATGCTGAAGAA	486
	R: CCTGCTGGAGCTGTTAGGT	
PGL3-basic-P45-MT	F: AGGGTCACTATGCAACCTAACAGCTCCAGCAGGTA	3178
	R: CTGTTAGGTTGCATAGTGACCCTCCTATGGGCCTT	
PGL3-basic-P56-WT	F: GCCAGGAGACAGGTTTTA	319
	R: GCAGTACGTGACTTCAGGA	
PGL3-basic-P56-MT	F: ATTTAATCATGGCATAGGTCAATGTTTGCAAAGTT	3011
	R: ATTGACCTATGCCATGATTAAATGTTCTCTAGAGG	
MyoG-CDS	F: CCGCTCGAGGCCACCATGGAGCTCTTTAGA	681
	R: CGGGGCCCACTTGGAAACAGCCACATTG	

*Sequences with underline represent the enzyme cutting sites.*

MiR-205a promoter reporter plasmid: Luciferase reporter vectors including different sized miR-205a promoter fragments were constructed from the chicken genome using NheI and XhoI restriction sites. The PCR products were excised with NheI and XhoI restriction endonucleases and cloned into plasmid vector pGL3-Basic (Promega, Fitchburg, WI, United States). The recombinant constructs were named pGL3-basic-P1 (−854/−127), pGL3-basic-P2 (−1447/−127), pGL3-basic-P3 (−1698/−127), pGL3-basic-P4 (−2133/−127), pGL3-basic-P5 (−2654/−127), pGL3-basic-P6 (−3026/−127), pGL3-basic-P7 (−3350/−127). These were numbered relative to the first base of the pre-miR-205a. The PCR products of the potential promoter region were ligated into the plasmid vector pGL3-Basic, which were named as pGL3-basic-P23 (−1698/−1447), pGL3-basic-P45 (−2654/−2133), and pGL3-basic-P56 (−3026/−2654). The predicted transcription factor binding sites were successfully mutated from TTACC to ACGGT for pGL3-basic-P23 vector, GCCAG to ATGCA for pGL3-basic-P45 vector and from AACAT to TGGCA for pGL3-basic-P56 vector. The major primers used for vector construction are listed in **Table 1**.

### Cell Culture

Quail muscle clone 7 cell lines were cultured in M199 medium (Gibco, United States) with 10% fetal bovine serum (FBS) (Gibco, United States), 10% tryptose phosphate broth solution (Sigma Life Science, United States), and 0.5% penicillin/streptomycin (Invitrogen, United States).

DF-1 cell lines were cultured in DMEM (Gibco, United States) with 10% FBS and 0.5% penicillin/streptomycin.

Chicken primary myoblast were isolated from the leg muscle tissues of E11 chickens. The muscle tissues were digested with trypsin for 10 min after the skin and bones were removed. After neutralization with complete medium, the cells were then collected by centrifugation at 1000 × *g* for 5 min. The differential attachment was used here to eliminate fibroblasts. Growth medium (GM) for primary myoblasts contained Roswell Park Memorial Institute (RPMI)-1640 medium (Gibco, United States) with 20% FBS and 0.5% penicillin/streptomycin. To induce myogenic differentiation, GM was replaced by differentiation medium (DM) containing PRMI-1640 with 5% FBS and 0.5% penicillin/streptomycin after CPM cells reached 80∼90% confluence.

### Cell Transfection

All the RNA oligonucleotides in this study {miR-205a mimics, miR-205a inhibitor, and si-CDH11 [small interfering RNA (siRNA) used for the knockdown of *CDH11*]} were obtained from RiboBio (Guangzhou, China) and the sequences are showed in **Table 2**. The transfection concentration of these three oligonucleotides was 20, 50, and 150 nM, respectively. Transfections were performed with Lipofectamine 3000 reagent (Invitrogen, United States) following the manufacturer’s protocol with at least three replications.

**Table 2:** Oligonucleotide sequences in this study.

Fragment name	Sequences (5′ to 3′)
miR-205a mimic	UCCUUCAUUCCACCGGAGUCUG
miR-205a inhibitor	UGGUUGUUUGGUGGCCUCUGUC
si-CDH11	CCACAAGCAACACCAGGAA

### RNA Isolation, Complementary DNA (cDNA) Synthesis, and Quantitative Real-Time PCR (qRT-PCR)

The total RNA was isolated from muscle tissues or cells using Trizol reagent (Takara, Japan). The reverse transcription reaction for mRNA was carried out using PrimeScript RT reagent Kit with gDNA Eraser (Perfect Real Time) (Takara, Japan) following the manufacturer’s instructions. cDNA synthesis for miRNA was performed with ReverTra Ace qPCR RT Kit (Toyobo, Japan) using specific Bulge-loop miRNA qRT-PCR Primer for miR-205a and U6 designed by RiboBio (Guangzhou, China). With iTaq Universal SYBR Green Supermix Kit (Toyobo, Japan), qRT-PCR reactions were carried out in a Bio-Rad CFX96 Real-Time Detection system (Bio-Rad, Hercules, CA, United States), and the data analysis was carried out with the comparative 2^−ΔΔC^_T_ method (Livak and Schmittgen, 2001). The major primers used for qRT-PCR are listed in **Table 3**.

**Table 3:** Primers used for quantitative real-time PCR (qRT-PCR).

Gene name	Primer sequences (5′ to 3′)	Size (bp)
*CDH11*	F: CCAACACCCTGACCATCC	187
	R: GGTTCTTTCTTCTGCCTTT	
*MyoD*	F: GCTACTACACGGAATCACCAAAT	200
	R: CTGGGCTCCACTGTCACTCA	
*MyoG*	F: CGGAGGCTGAAGAAGGTGAA	320
	R: CGGTCCTCTGCCTGGTCAT	
*MyHC*	F: CTCCTCACGCTTTGGTAA	213
	R: TGATAGTCGTATGGGTTGGT	
β*-actin*	F: GATATTGCTGCGCTCGTTG	194
	R: TTCAGGGTCAGGATACCTCTTT	

### Dual-Luciferase Reporter Assay

Wild-type or mutant CDH11-3′UTR dual-luciferase reporter (100 ng) and miR-205a mimic or NC duplexes (50 nM) were co-transfected into DF-1 cells in a 96-well plate. For the promoter assays, the reporter plasmid and the TK-Renilla reporter were co-transfected into DF-1 cells with six independent repeats. Firefly and Renilla luciferase activities were measured after 48 h transfection using a Dual-GLO Luciferase Assay System Kit (Promega, United States) in a Fluorescence/Multi-Detection Microplate Reader (BioTek, United States).

### Flow Cytometric Analysis

Quail muscle clone 7 or CPM cells cultured in 12-well plates were collected and fixed in 70% ethanol overnight at −20°C after 48 h transfection. Flow cytometric analysis was performed with a Cell Cycle Analysis Kit (Thermo Fisher Scientific, United States) on a BD Accuri C6 flow cytometer (BD Biosciences, United States). The data was processed using the FlowJo7.6 software.

### EdU Assay

Quail muscle clone 7 or CPM cells cultured in 24-well plates were incubated with 50 μM 5-ethynyl-2′-deoxyuridine (EdU; RiboBio) for 2 h at 37°C after 48 h transfection. The cells then were fixed and stained with a C10310 EdU Apollo In Vitro Imaging Kit (RiboBio, China). A Leica DMi8 fluorescent microscope was used to capture three randomly selected fields to visualize the EdU-stained cells. The proliferation rate was the ratio of the number of EDU-stained cells to the number of Hoechst 33342-stained cells.

### Immunofluorescence

Chicken primary myoblast cells cultured in 12-well plates were treated with 4% formaldehyde for 20 min after 48 h transfection and then permeabilized by 0.1% Triton X-100. Subsequently, the cells were blocked with goat serum for 30 min and incubation with anti-MyHC (B103; DHSB, United States; 0.5 μg/ml) overnight. After washed with PBS, the cells were treated with Fluorescein (FITC)-conjugated AffiniPure Goat Anti-Mouse IgG (H+L) (BS50950; Bioworld, United States; 1:50) for 1 h. The cell nuclei were stained with DAPI (Beyotime, China) for 10 min and then captured with Leica DMi8 fluorescent microscope. ImageJ software (National Institutes of Health) was used to measure the percentage of the total image area covered by myotubes.

### Western Blot Analysis

Chicken primary myoblast cellular proteins were extracted by using radio-immunoprecipitation assay (RIPA) buffer and phenylmethanesulfonyl fluoride (PMSF) protease inhibitor. Western blot assays were performed as previously reported (Bass et al., 2017). The antibodies used were as follows: rabbit anti-OB Cadherin (ab151302; Abcam, United States; 1:1000), goat anti-rabbit IgG-HRP (BA1055; Boster, China; 1:2500), rabbit anti-GAPDH (AB-P-R 001; Goodhere, China; 1:1500).

### Statistical Analysis

All experimental results are presented as mean ± SEM with at least three independent replications. The statistically significant difference between groups was tested by independent sample *t*-test.

## Results

### CDH11 Facilitates the Proliferation of Myoblast

**FIGURE 1:**
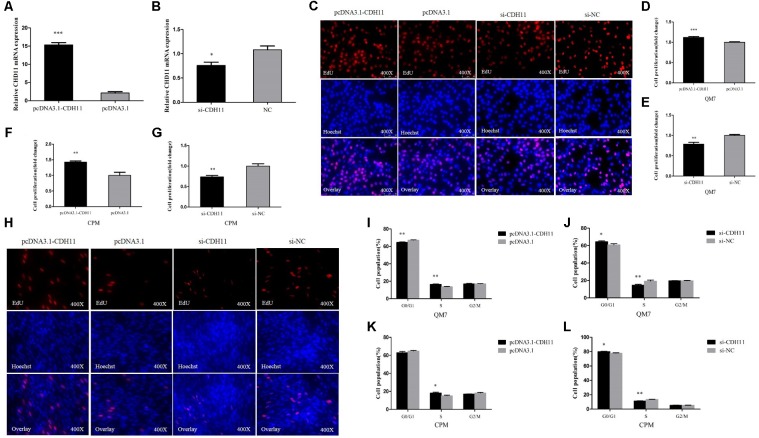
CDH11 facilitates the proliferation of myoblast. **(A,B)** The mRNA level of *CDH11* after *CDH11* overexpression and knockdown in CPM cells. **(C,H)** EdU proliferation assays for QM7 and CPM cells with overexpression and inhibition of CDH11. **(D–G)** The number of proliferative cells was also counted. **(I–L)** QM7 and CPM cells were collected for cell cycle analysis 48 h after transfection. Results are shown as mean ± SEM and the data are representative of at least three independent assays. Independent sample *t*-test was used to analyze the statistical differences between groups. ^∗^*P* < 0.05; ^∗∗^*P* < 0.01; ^∗∗∗^*P* < 0.001.

*Cadherin-11* was successfully overexpressed and knocked down in QM7 cells (**Figures 1A,B**). In QM7 cells, EdU-staining assay showed that the proliferation rate was significantly promoted when *CDH11* overexpression compared with that of the control cells, whereas *CDH11* loss-of-function by siRNA reduced cell proliferation rate (**Figures 1C–E**).

The EdU assay was also performed in CPM cells and the results were consistent with QM7 cells (**Figures 1F–H**). In cell cycle analysis, *CDH11* overexpression resulted in a decline in G0/G1 phase cells and an increased number of cells that progressed to S phase in both QM7 and CPM cells (**Figures 1I,K**). Conversely, the knockdown of *CDH11* can increase cell population in the G0/G1 phase and decrease cell population in the S phase in both QM7 and CPM cells (**Figures 1J,L**). Together, these results meant CDH11 can facilitate the proliferation of myoblast.

### CDH11 Is an Inhibitor of Myoblast Differentiation

**FIGURE 2:**
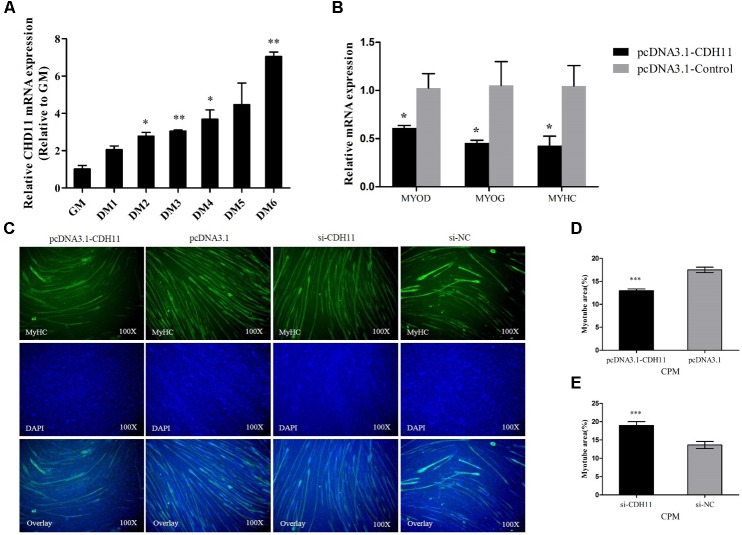
CDH11 is an inhibitor of myoblast differentiation. **(A)** The relative mRNA level of CDH11 during CPM cells differentiation. GM representing myoblasts were in proliferative phase, while DM1......DM6 means myoblasts were successfully induced to differentiate from day 1 to day 6. **(B)** The mRNA levels of myoblast differentiation marker genes were detected after transfected with pcDNA3.1-CDH11. **(C)** MyHC staining of CPM cells at 72 h after transfected with pcDNA3.1-CDH11 and si-CDH11. **(D,E)** Myotube area (%) of CPM cells with *CDH11* overexpression and knockdown. Results are shown as mean ± SEM and the data are representative of at least three independent assays. Independent sample *t*-test was used to analyze the statistical differences between groups. ^∗^*P* < 0.05; ^∗∗^*P* < 0.01; ^∗∗∗^*P* < 0.001.

To further investigate the potential roles of CDH11 in myoblast differentiation, CPM cells were induced to differentiate *in vitro*. We found that the expression of CDH11 is gradually increased with the process of differentiation (**Figure 2A**). Meanwhile, the overexpression of *CDH11* could inhibit the expression of MyoD, MyoG, MyHC (**Figure 2B**) which are known as myoblast differentiation marker genes. Immunofluorescence staining showed that *CDH11* overexpression inhibited myoblast differentiation by increasing total areas of myotubes (**Figures 2C,D**), while the knockdown of *CDH11* could promote myoblast differentiation (**Figures 2C,E**). All these results suggested that CDH11 is an inhibitor of myoblast differentiation.

### CDH11 Is a Direct Target of MiR-205a

**FIGURE 3:**
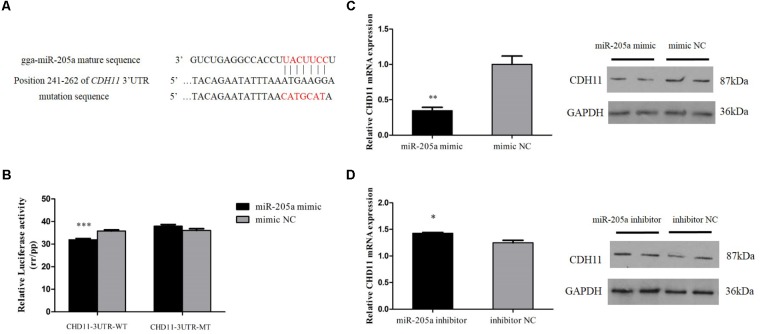
CDH11 is a direct target of miR-205a. **(A)** The miR-205a binding site in the *CDH11* mRNA 3′UTR. The seed sequence and mutant sequence in miR-205a is highlighted in red. **(B)** The dual-luciferase reporter assay was performed by co-transfecting wild-type or mutant CDH11 3′UTR with miR-205a mimic of mimic NC in DF-1 cells. **(C,D)** The mRNA and protein level of CDH11 from miR-205a mimic and inhibitor transfected CPM cells. Results are shown as mean ± SEM and the data are representative of at least three independent assays. Independent sample *t*-test was used to analyze the statistical differences between groups. ^∗^*P* < 0.05; ^∗∗^*P* < 0.01; ^∗∗∗^*P* < 0.001.

The miR-205a binding site in the 3′UTR region of CDH11 had been mutated successfully (**Figure 3A**). The recombinant reporter vectors (pmirGLO-CDH11-WT and pmirGLO-CDH11-MT) were co-transfected with miR-205a mimic or mimic negative control (NC) and then a dual-luciferase reporter assay was carried out in embryonic chicken fibroblast cell line DF-1 cells. Mir-205a mimics were able to reduce the luciferase activity compared with the NC group after co-transfected with pmirGLO-CDH11-WT, whereas no significant difference was observed in the mutant group (pmirGLO-CDH11-MT) (**Figure 3B**). The mimic and inhibitor of miR-205a were transfected into CPM cells, respectively, and qRT-PCR and Western blot found that miR-205a could significantly decrease the expression of CDH11, while inhibition of miR-205a up-regulates CDH11 expression (**Figures 3C,D**). These results indicated that CDH11 is a direct target of miR-205a.

### MiR-205a Represses the Proliferation of Myoblast

**FIGURE 4:**
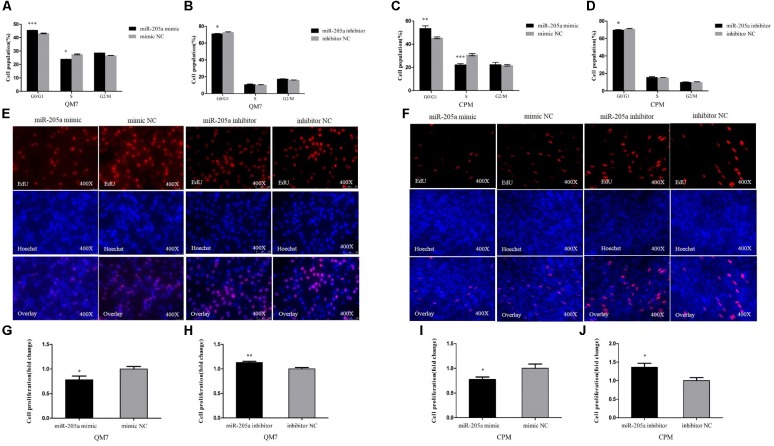
MiR-205a represses the proliferation of myoblast. **(A–D)** Cell cycle analysis of QM7 and CPM cells 48 h after overexpression and inhibition of miR-205a. **(E,F)** EdU staining after transfection of miR-205a mimic and miR-205a inhibitor in QM7 and CPM cells. **(G–J)** The proliferation rate of QM7 and CPM cells transfected with miR-205a mimic and inhibitor. Results are shown as mean ± SEM and the data are representative of at least three independent assays. Independent sample *t*-test was used to analyze the statistical differences between groups. ^∗^*P* < 0.05; ^∗∗^*P* < 0.01; ^∗∗∗^*P* < 0.001.

In order to unveil the functions of miR-205a, overexpression and inhibition experiments were performed to assess its role in cell proliferation. In QM7 cell lines, we found that the overexpression of miR-205a could significantly increase the number of cells in G0/G1 phase, and the number of cells remaining in S phase was lower than that in control group (**Figure 4A**), in the meantime, the cell cycle changes transfected with miR-205a inhibitor showed the opposite trend (**Figure 4B**). The EdU assay (**Figure 4E**) and the statistics of cell proliferation rate (**Figures 4G,H**) showed that miR-205a could hamper the proliferation of QM7 cells. The same results were also found in CPM (**Figures 4C,D,F,I,J**) demonstrating that miR-205a could repress the proliferation of myoblast.

### MiR-205a Promotes the Differentiation of Myoblast

**FIGURE 5:**
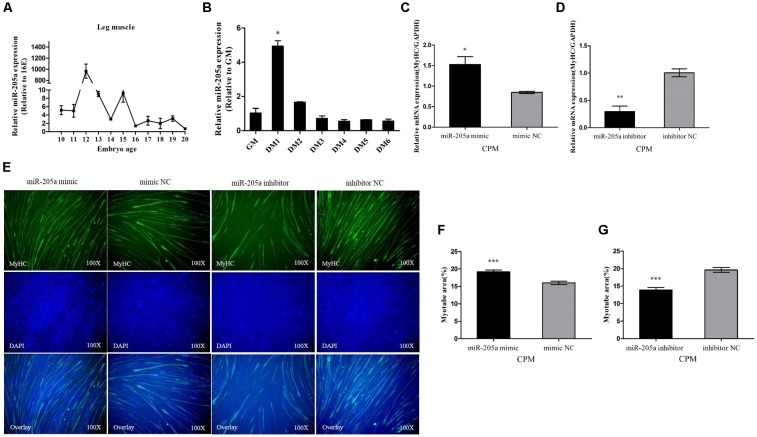
MiR-205a promotes the differentiation of myoblast. **(A)** The relative expression of miR-205a in chicken embryonic leg muscle. **(B)** The relative expression of miR-205a during CPM differentiation. GM representing myoblasts in proliferative phase, while DM1......DM6 means myoblasts successfully induced to differentiate from day 1 to day 6. **(C,D)** The mRNA level of MyHC from miR-205a mimic and inhibitor transfected CPM cells. **(E)** MyHC staining of CPM cells at 72 h after transfection of miR-205a mimic and inhibitor. **(F,G)** Myotube area (%) of CPM cells 72 h after overexpression and inhibition of miR-205a. Results are shown as mean ± SEM and the data are representative of at least three independent assays. Independent sample *t*-test was used to analyze the statistical differences between groups. ^∗^*P* < 0.05; ^∗∗^*P* < 0.01; ^∗∗∗^*P* < 0.001.

During skeletal muscle development in Xinghua chickens, miR-205a abruptly showed an extremely high expression at 12E which indicated its potential involvement in skeletal muscle differentiation (**Figure 5A**). CPM cells were induced to differentiate *in vitro*, and the miR-205a had a relatively high expression when CPM cells starting differentiation (**Figure 5B**). Therefore, we transfected miR-205a mimic and inhibitor into CPM cells. Overexpression of miR-205a remarkably increased the expression of MyHC, whereas the knocking down of miR-205a decreased the expression of MyHC (**Figures 5C,D**). In addition, miR-205a can promote the formation of myotubes (**Figures 5E–G**). All these results stated clearly that miR-205a could promote the differentiation of myoblast.

### MyoG Could Regulate the Expression of MiR-205a

**FIGURE 6:**
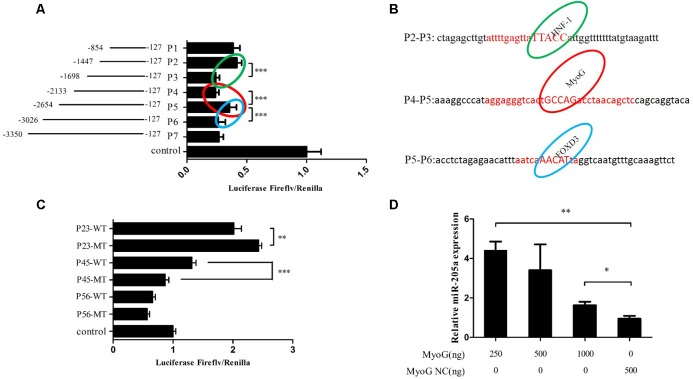
MyoG could regulate the expression of miR-205a. **(A)** Dual-luciferase reporter assay using the clones’ upstream regions of the chicken pre-miR-205a. **(B)** Three possible transcription factors predicted on the Gene-regulation. Their binding sites are highlighted in the red capital. **(C)** Luciferase assay was conducted by co-transfecting the vectors containing potential promoter region or mutant transcription factors binding sites with TK-Renilla plasmid. **(D)** Overexpression of *MyoG* can promote miR-205a expression. Results are shown as mean ± SEM and the data are representative of at least three independent assays. Independent sample *t*-test was used to analyze the statistical differences between groups. ^∗^*P* < 0.05; ^∗∗^*P* < 0.01; ^∗∗∗^*P* < 0.001.

A series of individual segments were cloned into the pGL3-Basic vector to further analysis the promoter activity region of miR-205a. Results showed that the luciferase activities were different between three regions, P2 and P3, P4 and P5, P5 and P6 (**Figure 6A**). Therefore, we predicted possible transcription factors that could bind to these three areas on the Gene-regulation^1^ database (**Figure 6B**). The transcription factor binding sites were successfully mutated and cloned into pGL3-Basic vector along with the promoter activity regions. Dual-luciferase reporter assay results showed that the mutation of HNF-1 binding sites could increase the luciferase activity while the mutated MyoG binding sites decrease the luciferase activity (**Figure 6C**). Also, the overexpression of *MyoG* can up-regulate the expression of miR-205a (**Figure 6D**). Taken together, these results demonstrated that MyoG can regulate the expression of miR-205.

## Discussion

In this study, we reported a role for miR-205a in myoblast proliferation and differentiation by repressing CDH11 that can promote proliferation and suppress differentiation. In addition, MyoG can regulate the expression of miR-205a by binding to the gga-miR-205a gene promoter activity region.

Each miRNA could have hundreds of target genes (Lewis et al., 2005), so does miR-205a. Previous research showed that the miR-200 family and miR-205 can regulate the transformation of epithelial-mesenchymal transition (EMT) by involving in the TGF-β signaling pathway and targeting ZEB1 and ZEB2 (Gregory et al., 2008). Also, there is evidence that miR-205 can inhibit EMT by targeting ZEB2 (Matsushima et al., 2011). It is reported that ZEB1 and E2F1 can inhibit the differentiation of primary myoblasts, besides, E2F1 can also promote myoblast proliferation which was reported as a putative target gene of miR205 (Gandellini et al., 2009; Luo et al., 2016; Li et al., 2017). So there is a high possibility that miR-205a could hamper myoblast proliferation by involving in the TGF-β signaling pathway and targeting E2F1 and facilitate primary myoblast differentiation by repressing ZEB1 and E2F1. MiR-205 can widely participate in cancer development and was once defined as esophageal squamous cell carcinoma-specific miRNA (Greene et al., 2010; Matsushima et al., 2011). However, in the current study, we found that miR-205a has a transient high expression at E12, E13, and E15 during chicken leg muscle development, which means miR-205a can also be expressed in early skeletal muscle cells and have functions in muscle development besides cancer cells.

Early studies showed that CDH2 and CDH11 can transfer the intercellular adhesion force to the actin stress fiber network thus increasing the contraction of acting and promoting wound closure during the transformation of fibroblasts into myofibroblasts (Hinz and Gabbiani, 2003; Hinz et al., 2004). Another report showed that CDH11 could affect SMC differentiation by regulating the expression of TGF-β1 and participating in TGFβRII pathway (Alimperti et al., 2014). So we have reasons to believe that both miR-205a and CDH11 can play their roles in myoblast proliferation and differentiation through TGF-β signaling pathway. But the validation experiment still needs to be done to verify our inference.

**FIGURE 7:**
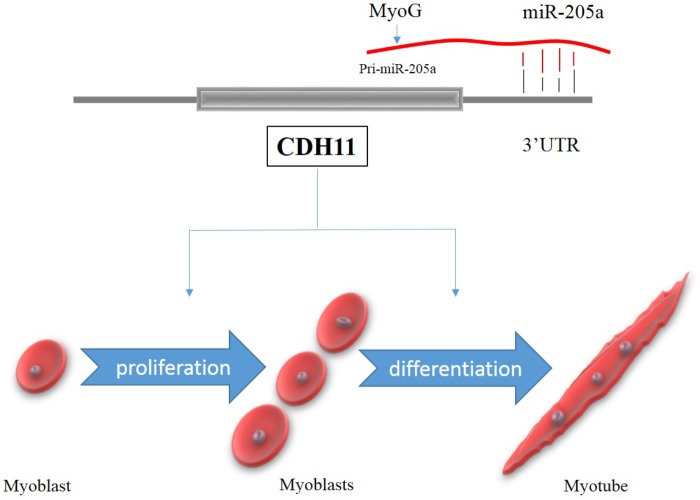
A schematic model of CDH11 mediated regulatory network during myoblast proliferation and differentiation.

The expression of miRNAs can be regulated by specific transcription factors (Markopoulos et al., 2018). In this study, we found two candidate transcription factors MyoG and HNF-1 that could bind to the promoter activity regions and regulate the expression of miR-205a. The luciferase activity of P1 to P7 was all lower than the control group (**Figure 6A**), which means the segment in pGL3-basic-P1 vector have the existence of silencer since the luciferase activity of P23 and P45 were higher than control (**Figure 6C**). Research also found that ErbB2 can negatively regulate the expression of miR-205 in breast cancer (Adachi et al., 2011). MyoG, a key regulator of muscle development can regulate the transcription of most of the muscle-specific genes (Cao et al., 2006; Braun and Gautel, 2011; Luo et al., 2015) and is essential for the terminal differentiation of myoblasts (Edmondson and Olson, 1990). HNF-1 is a transcription factor widely expressed in many tissues and cell types, which can be highly expressed in the liver and involved in the regulation of the expression of a variety of liver-specific genes (Courtois et al., 1987). Our data indicated that the transcription factor MyoG could regulate the expression of miR-205a. But it still remains to be determined how HNF-1 can regulate miR-205a. However, based on our findings, miR-205a plays an essential role in myoblast proliferation and differentiation by targeting CDH11. In this study we proposed a schematic model of CDH11 mediated regulatory network during myoblast proliferation and differentiation (**Figure 7**).

## Conclusion

It was demonstrated that MiR-205a was regulated by MyoG transcription factor and the up-regulation of miR-205a can inhibit myoblast proliferation and promote myoblast differentiation by its repression on CDH11.

## Author Contributions

ZW designed the study, carried out all experiments, analyzed the data, and wrote the paper. HO participated in the design of the experiments and data analysis. XC participated in the data collection and interpretation. JY and BC helped with performing some of the manuscripts’ experiments. BA helped for useful discussion and language correction. QN participated in the design, manuscript writing, and final approval of the manuscript. All authors read and approved the final manuscript.

## Conflict of Interest Statement

The authors declare that the research was conducted in the absence of any commercial or financial relationships that could be construed as a potential conflict of interest.

## Footnotes

1 http://gene-regulation.com/
